# Performance-Related and Body Composition Differences Between High and Low-Performing Taekwondo Athletes: An Exploratory Study

**DOI:** 10.3390/jfmk11030342

**Published:** 2026-08-31

**Authors:** Angeliki Kavvoura, Nikolaos Zaras, Evangelia Makri, Christos Ormanlidis, Alexandra Avloniti, Asimenia F. Gioftsidou, Athanasios Chatzinikolaou, Anastasia Beneka, Gerasimos Terzis, Vivian Paraskevi Malliou

**Affiliations:** 1 Department of Physical Education and Sport Science, School of Physical Education, Sport Science and Occupational Therapy, Panepistimioupoli Campus, Democritus University of Thrace, 96100 Komotini, Greece; akavvour@phyed.duth.gr (A.K.); emakri@phyed.duth.gr (E.M.); chormanl@phyed.duth.gr (C.O.); alavloni@phyed.duth.gr (A.A.); agioftsi@phyed.duth.gr (A.F.G.); achatzin@phyed.duth.gr (A.C.); ampeneka@phyed.duth.gr (A.B.); pmalliou@phyed.duth.gr (V.P.M.); 2 School of Physical Education and Sport Science, National and Kapodistrian University of Athens, Ethnikis Antistasis 41, Daphne, 17237 Athens, Greece; gterzis@phed.uoa.gr

**Keywords:** repeated kicks, lean mass, power, knee extension, flexion torque

## Abstract

**Objectives**: The main purpose of this exploratory study was to examine differences between high-performing (HP) and low-performing (LP) female taekwondo athletes in body composition, lower extremities power, and knee extension/flexion isokinetic torque. A secondary aim was to explore how body composition, torque, and power relate to performance in the multiple frequency speed of kick test (FSKT_MULT_). **Methods**: Twenty-six female taekwondo athletes (age: 18.0 ± 2.8 years; body mass: 58.1 ± 9.9 kg; height: 166.0 ± 0.05 cm) participated in the study. Athletes were classified as high performers (HP) or low performers (LP) based on their total score in the FSKT_MULT_. Measurements included body composition assessed with dual-energy X-ray absorptiometry, countermovement jump (CMJ), standing long jump (LJ), and isokinetic knee extension and flexion at 60°/s and 240°/s. **Results**: High performers completed significantly more kicks during the first FSKT_MULT_ set than low performers (19.8 ± 1.1 vs. 18.0 ± 0.9 kicks; *p* = 0.001). They also showed higher trunk lean mass (*p* = 0.038), greater total lean mass (*p* = 0.036), and better standing long jump performance (*p* = 0.016). Mean and peak knee-flexion torque at 60°/s were higher in HP compared to LP. When all athletes were analyzed as one group, moderate-to-large correlations were observed between FSKT_MULT_ performance and body composition, as well as knee extension and flexion torque at both 60°/s and 240°/s. **Conclusions**: These findings indicate that high-performing female taekwondo athletes are stronger and generate greater power than low performers. Lean mass and knee extension and flexion torque showed moderate associations with FSKT_MULT_ performance.

## 1. Introduction

Taekwondo is considered a high intermittent combat sport in which lower extremities activation appears to have the dominant role in match outcome [1]. During a competition, the bandal chagui is the most frequently used kicking technique [2,3,4]. In recent years, this technique has been used to develop the multiple frequency speed of kick test (FSKT_MULT_), an anaerobic test consisting of five 10 s sets separated by 10 s rest intervals [5]. The FSKT_MULT_ provides information about the anaerobic profile of taekwondo athletes by measuring the highest number of kicks achieved in 10 s (FSKT_BEST_), the total number of kicks performed during the entire test (FSKT_TOTAL_), and the percentage kick decrement index (FSKT_%KDI_), which reflects the rate of performance decline [5]. The FSKT_MULT_ has been used to classify male and female athletes [6], and evidence from 41 female taekwondo athletes suggests that FSKT_MULT_ appeared to be an effective tool to discriminate the competitive level from international/national to state/region level athletes. Specifically, international/national level athletes achieved higher FSKT_BEST_ as well as a higher FSKT_TOTAL_ [4]. Similarly, the study of Moreira et al. [7] reported significant differences between elite and sub-elite athletes in bandal-chagui performance, with elite athletes demonstrating higher angular velocities, greater linear velocities, and faster timing parameters. However, the study investigated the bandal chagui kicking technique exclusively, and the discrimination of elite and sub-elite athletes was performed following nine maximum velocity kicks with 15 s rest. Because FSKT_MULT_ requires repeated high velocity kicking, it may better differentiate athletes with higher strength, power, and lean mass, while also providing coaches with valuable information regarding fitness conditioning. These premises, however, require further investigation.

Taekwondo athletes compete in distinct body-weight categories, meaning that increases in lean mass—and especially in body fat—may influence total body mass and potentially alter an athlete’s competitive category. Because FSKT_MULT_ performance depends on repeated high-velocity kicking, physical characteristics such as lean mass and body fat may influence an athlete’s ability to sustain forceful kicks. Although lean mass is a significant contributor to high-level performance in strength and power athletes [8], the correlation between lean mass and FSKT_MULT_ is unclear. Ferreira da Silva Santos et al. [6] showed that the weight category did not seem to differentiate performance in FSKT_MULT_ in 70 female taekwondo athletes. However, body fat was negatively correlated with FSKT_MULT_ (r = −0.606; *p* = 0.006) in a group of sixteen male taekwondo athletes, although body composition was evaluated with skinfolds [9]. Additionally, asymmetries in fat-free mass and lean soft tissue mass predicted FSKT_%KDI_ by 21%, and bone mineral density asymmetry negatively affected FSKT_MULT_ performance by 48% in a mixed-gender sample of seventeen athletes [10]. A recent study also found significant correlations between lean mass and FSKT_MULT_ performance (r ranged from 0.369 to 0.613), though again in a mixed-gender sample [11]. Whether body composition variables—particularly lean mass—are associated with FSKT_MULT_ performance in female athletes remains unclear. Furthermore, it is unknown whether high performers in FSKT_MULT_ possess greater lean mass than lower-performing counterparts.

Kicking performance in taekwondo depends on several factors, including joint mobility, flexibility, reaction time and the interaction between agonist and antagonist muscles, all of which influence kick angular velocity [7,12]. Since lean mass contributes to force production, and force production directly affects kicking velocity, strength and power characteristics may also play a crucial role in FSKT_MULT_ performance. Maximum strength and especially the rate of force development are essential for maximizing movement velocity in taekwondo athletes [13]. Although lean mass has shown moderate correlations with FSKT_MULT_, recent evidence suggests that strength is also a significant contributor to kicking performance. Hip muscle strength appears to be the dominant muscular factor determining kicking performance, while knee angular velocity combined with hip torques may best discriminate elite athletes from sub-elite athletes [14]. However, these findings are based solely on bandal chagui performance (9 kicks with 15 s rest) and the relationship between strength/power and FSKT_MULT_ performance remains largely unclear. Therefore, it would be interesting to investigate whether high-performance taekwondo athletes may differentiate lower-extremity strength and power compared to lower-performance athletes.

The purpose of the study was to investigate the differences between high and low performers in FSKT_MULT_ in body composition and lower-extremity torque and power production, and to explore correlations between FSKT_MULT_ and body composition, force and power variables in female taekwondo athletes. It was hypothesized that high-performing athletes would exhibit greater lean mass, force, and power, and that FSKT_MULT_ performance would correlate with body composition, force, and power.

## 2. Materials and Methods

### 2.1. Subjects

Twenty-six well-trained female taekwondo athletes [age:18.0 ± 2.8 years (age ranged from 14 to 22 years); body mass: 58.1 ± 9.9 kg; body height: 166.0 ± 0.05 cm; training experience: 10.1 ± 3.7 years] gave their written consent to participate in the study after being informed about the experimental procedures. Athletes were recruited after personal communication with coaches. Parental consent was also obtained for athletes under 18 years of age. All athletes were black belt with at least 1 Dan and participated in competitions during the study. The inclusion criteria for participating in the study were: (a) trained on a regular basis (≥5 training sessions per week), (b) participated in the national championship during the current year when the study was conducted, (c) absence of any orthopedic or neuromuscular issues and (d) absence of any drug or illegal substances. All procedures were in accordance with the 1975 Declaration of Helsinki as revised in 2000 and were approved by the Institutional Ethics Committee of the local university (protocol number: ΔΠΘ/EHΔE/5603/75).

### 2.2. Experimental Design

This is a cross-sectional study. Female taekwondo athletes participated in a 3-day experimental procedure. During the first day, anthropometric characteristics and body composition analysis were performed. Then, a familiarization training session with countermovement jump (CMJ), standing long jump (LJ) and isokinetic knee extension/flexion was performed. During the second day, measurements of CMJ and LJ were performed while the FSKT_MULT_ on a standing mannequin was also measured. During the third day of measurements, athletes performed the isokinetic knee extension and flexion at 60° and 240°. Following the measurements, athletes were divided into high performers (HP; N = 13) and low performers (LP; N = 13) according to the median value of the FSKT_TOTAL_ kick performance [15,16]. This classification was made since the purpose of the current exploratory study was to investigate whether performance in FSKT_TOTAL_ may discriminate female taekwondo athletes in body composition and lower-extremity torque and power production. Comparisons were made between groups while correlations were performed including all athletes. During all measurements, athletes were between the follicular and ovulation phases and in good shape to perform.

### 2.3. Body Composition and Familiarization Session

During the first day, anthropometric characteristics and body composition analysis were evaluated. Participants reported at the laboratory during morning hours in a fasted state for the evaluation of their body composition through Dual-Energy X-Ray Absorptiometry (DEXA) (Lunar iDXA, GE Healthcare, Madison, WI, USA). Athletes were asked to abstain from strenuous physical activities for at least 24 h before the evaluation. All DEXA scans and region-of-interest analyses were performed by a trained single operator who received documented, supervised practical training in DEXA acquisition and region-of-interest analysis from the board-certified radiologist responsible for the scientific oversight of the DEXA unit as well as radiation protection training from the nuclear physicist responsible for radiation protection matters at the facility. The enCORE software, version 14.10.022 (GE Healthcare, Madison, WI, USA), was used for the whole-body and segmental body composition analysis module for the determination of body fat (%), total lean mass, upper extremities lean mass, lower extremities lean mass and trunk lean mass. Body height was measured using a portable stadiometer (HM202P, Charder Electronic Co., Ltd., Taichung City, Taiwan) and body mass was measured on a portable body scale (Tanita BC-545n, Southampton, UK). The intraclass correlation coefficients (ICC) for all variables are presented in previous studies [17,18]. Following the anthropometric and body composition measurements, athletes performed a familiarization session to become accustomed to the laboratory testing procedures. After a 5 min warm-up on a stationary bicycle at approximately 70–80 rpms athletes performed 2 sets of 15 s static stretching, including upper body chest and triceps stretching and quadriceps, hamstrings, gluteus and calves stretching. Then, athletes performed 2 sets of 10 s dynamic stretching exercises, including shoulders forward and backward rotations, trunk side bending and twists, lower back flexion and extension and leg swings. Following the warm-up, athletes performed 5 submaximal and 5 high-intensity CMJs and LJs to familiarize themselves. Then athletes sat on the isokinetic dynamometer and performed several isokinetic attempts at velocities of 60° and 240°. Trials included 2–3 warm-up attempts with progressive application of force and 2 sets of 5 repetitions with force being applied as fast as possible. During all measurements, live images of the torque applied were offered to athletes and clarifications were given on how to achieve a higher performance. Athletes finished the familiarization session with a cool-down on a stationary bicycle and some light static stretching exercises.

### 2.4. Countermovement Jump and Long Jump

For the second day of measurements, athletes were instructed to refrain from any strenuous exercise for at least 24 h and be well-hydrated and fed. Measurements were performed during morning hours at a standard ambient temperature of 23 °C. The same warm-up procedure used during familiarization was performed prior to measurements, followed by 3 CMJs with submaximal intensity, with 1 min rest between jumps. Then, athletes performed 5 maximum CMJs, with 2 min rest between attempts. All CMJs were performed on a non-slider surface with arms akimbo using the Opto jump system (OptoJump Next, Microgate, Bolzano, Italy). Athletes were instructed to jump as high as possible, and following each jump, they were informed of their jump height, which served as motivation to jump higher [19]. From the CMJs, the maximum vertical jump height, power output and power relative to body mass during the push-off phase were analyzed. The best jumping height performance was used for statistical analysis. The ICCs for CMJ variables are presented elsewhere [20].

Following the CMJs, athletes performed the standing LJ. Athletes performed 3 LJs with submaximal intensity, with 1 min rest between attempts, and then athletes performed 5 maximum LJs, with 2 min rest between attempts. Long jumps were performed barefoot on the soft surface of taekwondo tatami. The distance was measured to the nearest centimeter from the take-off line to the mark where the heels landed on the floor via a metal measuring tape. The best LJ was used for the statistics. The ICC for LJ has been presented elsewhere [21].

### 2.5. Multiple Frequency Speed of Kick Test

Ten minutes following the standing LJ, athletes performed the FSKT_MULT_. The FSKT_MULT_ is a high-intensity combat test where athletes perform alternating kicks with the bandal chagui technique for 10 s with 10 s rest (ratio 1:1) for 5 sets on a standing mannequin or sandbag [5,22]. Athletes were familiar with the current test since they used it frequently under training conditions. Before the initiation of the test, the mannequin was adjusted to each athlete’s body height, and athletes had time to find their kicking distance from the targets. Athletes had the instruction to perform as many kicks as possible from the initiation of the test. If an athlete managed to perform more kicks than the first set in the remaining 4 sets, then the test was terminated and the athlete had to start again. From the FSKT_MULT_, the number of kicks during all sets (FSKT_TOTAL_), the number of kicks during the first set (FSKT_1SET_) and the percentage kick decrement index (FSKT_%KDI_) [9] were measured. The ICC for the FSKT_MULT_ has been presented elsewhere [21].

### 2.6. Isokinetic Knee Extension and Flexion

During the third day, the isokinetic knee extension and flexion were performed. Prior to the isokinetic evaluation, all athletes completed the Waterloo footedness questionnaire for the determination of the preferred leg [23]. After a five min warm-up on an ergometer (70–100 W), followed by 5 min of static and dynamic stretching of the lower extremities, including quadriceps/hamstrings and calves stretching and hip swings, athletes were seated on the dynamometer’s chair (Isoforce, TUR Gmbh, Berlin, Germany) by pelvic strapping with their back slightly reclined, their thighs well supported on the seat and their hands around the chest. The axis of rotation of the dynamometer was aligned with the femoral condyle of the knee. Also, the special lever arm was adjusted according to the length of the tibia and eventually adjusted above the ankle, over the medial malleolus. The test’s knee range of motion was defined as 0° (full knee extension) to 90°. Concentric mean torque (MT) and peak torque (PT) of knee extensors and flexors were measured. Testing included one session of five maximal knee extension/flexion repetitions at each tested speed (60°/s and 240°/s) [14], while exercising legs were randomly measured. Athletes recovered passively for two minutes between trials. The highest value tested for each velocity was recorded. Three minutes of rest were given for resetting the position and evaluating the other leg. The intra-class correlation coefficients have been presented in a previous study [24].

### 2.7. Statistical Analysis

The Shapiro–Wilk test revealed normal distribution for all the considered variables; Levene’s test for equality of variances was not significant, and all variables met the linearity assumption (*p* > 0.05). Therefore, all variables are presented with means and standard deviations (M ± Sd). An a priori power analysis [25] was used for the determination of the minimum sample size. G*Power 3 software (version 3.1.9.7, University of Dusseldorf, Dusseldorf, Germany) showed that for the comparison between groups, a total of 26 participants would be required for power = 0.720, d = 1.02, and an α-value of 0.05 [10,11,19]. In addition, for the correlational analysis, a bivariate normal model was used, two-tailed, α = 0.05, β = 0.10, and r = 0.60, in accordance with a previous study [10]. The comparison between HP and LP was performed with an independent samples T-Test. Hedges g effect size was also calculated. For the correlation between variables, the Pearson-r correlation coefficient was used. Hopkins scales were used to investigate and interpret the magnitude of effect for the correlations: trivial < 0.10; small < 0.10–0.29; moderate ≥ 0.30–0.49; large ≥ 0.50–0.69; very large ≥ 0.70–0.89; and nearly perfect ≥ 0.9 [26]. All statistical analyses were performed with SPSS-31, and the level of significance was set at *p* ≤ 0.05.

## 3. Results

Significant differences were found between HP and LP for FSKT_MULT_. As expected, a significant difference was found for the FSKT_TOTAL_ between groups (HP = 89.5 ± 3.2 kicks; LP = 79.0 ± 3.0 kicks; t = −8.668; *p* = 0.001; g = −3.292) since it was the variable that separated the two groups. For descriptive characterization of groups, HP had significantly higher performance in all sets compared to LP (set 1: HP =19.8 ± 1.1 kicks; LP = 18.0 ± 0.9 kicks; t = −4.737; *p* = 0.001; g = −1.799; set 2: HP = 19.2 ± 0.8 kicks; LP = 16.5 ± 1.1 kicks; t = −7.129; *p* = 0.001; g = −2.708; set 3: HP = 17.8 ± 0.8 kicks; LP = 15.7 ± 1.0 kicks; t = −5.947; *p* = 0.001; g = −2.259; set 4: HP = 17.1 ± 1.2 kicks; LP = 15.2 ± 1.0; t = −4.490; *p* = 0.001; g = −1.705; set 5: HP = 15.5 ± 1.1; LP = 13.8 ± 0.9; t = −4.554; *p* = 0.001; g = −1.730). However, no significant difference was found for FSKT_%KDI_ between groups (HP = −9.5 ± 3.2%; LP = −12.0 ± 3.7%; t = −1.795, *p* = 0.085; g = −0.682). Significant differences were found for body composition and LJ, but not for CMJ variables between HP and LP (Table 1).

**Figure 1:**
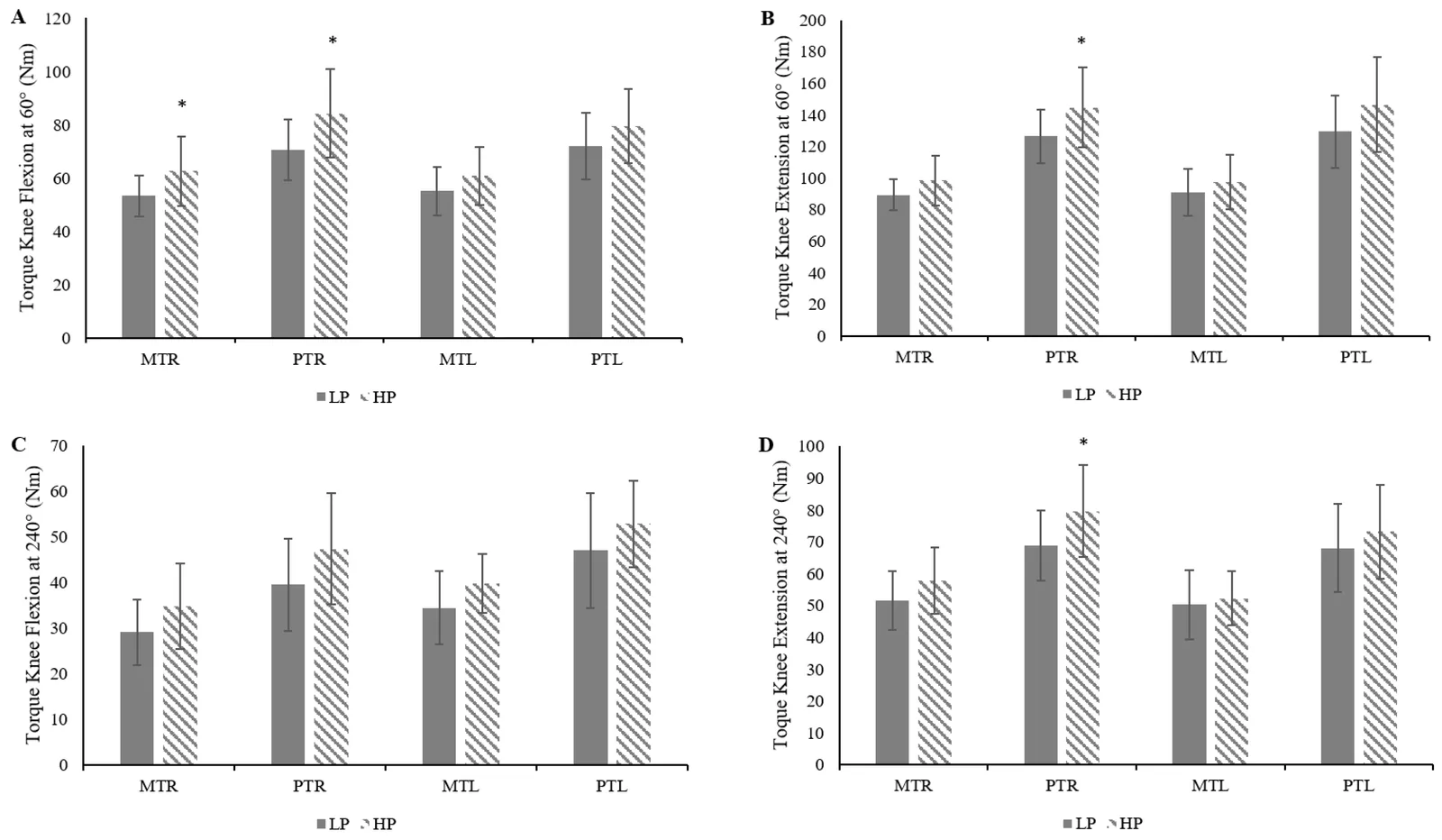
Comparison between high and low performers in isokinetic mean and peak torque during (**A**) knee flexion at 60°, (**B**) knee extension at 60°, (**C**) knee flexion at 240° and (**D**) knee extension at 240°. * = significant difference between high and low performers, LP = low performers, HP = high performers, MTR = mean torque right, PTR = peak torque right, MTL = mean torque left, PTL = peak torque left.

According to the results from the Waterloo footedness questionnaire, all athletes preferred the right leg. The analysis of the isokinetic knee extension and flexion showed significant differences between HP and LP (Figure 1). Specifically, significant difference was found for the right leg flexion at 60°/s (MT: t = −2.203, *p* = 0.037, g = −0.837; PT: t = −2.447, *p* = 0.022, g = −0.926) but not for the left leg flexion (MT: t = −1.466, *p* = 0.156, g = −0.557; PT: t = −1.430, *p* = 0.166, g = −0.543, Figure 1A). In addition, significant difference was found for the right knee PT extension at 60°/s (MT: t = −1.787, *p* = 0.087, g = −0.679; PT: t = −2.172, *p* = 0.040, g = −0.825) but not for the left knee extension (MT: t = −1.005, *p* = 0.325, g = −0.382; PT: t = −1.643, *p* = 0.113, g = −0.624, Figure 1B). No difference was found for the right knee flexion at 240°/s (MT: t = −1.749, *p* = 0.093, g = −0.664; PT: t = −1.779, *p* = 0.088, g = −0.676) and for the left knee flexion (MT: t = −1.869, *p* = 0.074, g = −0.710; PT: t = −1.341, *p* = 0.192, g = −0.509, Figure 1C). Significant difference was found for the right knee PT extension (MT: t = −1.623, *p* = 0.118, g = −0.617; PT: t = −2.128, *p* = 0.044, g = −0.808) but not for the left knee extension (MT: t = −0.518, *p* = 0.609, g = −0.197; PT: t = −0.902, *p* = 0.376, g = −0.342, Figure 1D).

Significant correlations were found between body composition variables and FSKT_MULT_ (Table 2). Specifically, small-to-large correlations were found between BMD and FSKT_MULT,_ while trivial-to-large correlations were found between lean mass and FSKT_MULT_. Additionally, fat percentage had only trivial-to-small correlations with FSKT_MULT_.

Table 3 presents the correlation coefficients between FSKT_MULT_ and isokinetic knee flexion and extension torque production during the 60° and 240°/s velocity trials. No significant correlations were found between CMJ variables and FSKT_MULT_ (r ranged from 0.008 to 0.339, trivial-to-moderate, *p* > 0.05). Additionally, statistical trends were observed for the correlations between LJ and FSKT_BEST_ (r = 0.374; *p* = 0.060; moderate) and with FSKT_TOTAL_ (r = 0.373; *p* = 0.061; moderate).

Significant correlations were found between CMJ power and total BMD (r = 0.437; *p* = 0.025; moderate), total lean mass (r = 0.808; *p* = 0.001; very large), trunk lean mass (r = 0.748; *p* = 0.001; very large) and legs lean mass (r = 0.825; *p* = 0.001; very large). No significant correlations were found between CMJ height and total BMD (r = 0.093; *p* = 0.648, trivial) and lean mass variables (r ranged from 0.073 to 0.280; *p* < 0.05; trivial-to-small). Lean mass variables were significantly correlated with CMJ power relative to mass (r ranged from 0.503 to 0.507, *p* = 0.009 to 0.001; large) while total BMD had a non-significant moderate correlation with CMJ relative to mass (r = 0.324; *p* = 0.106). Performance in LJ was significantly correlated with total BMD (r = 0.411; *p*= 0.037; moderate) but not with lean mass variables (r ranged from 0.105 to 0.327; *p* < 0.05; small-to-moderate). Correlations between body composition variables and isokinetic knee extension and flexion are presented in Table 4.

## 4. Discussion

The purpose of the study was to investigate the differences between HP and LP female taekwondo athletes in body composition, lower extremities power and knee extension and flexion torque production. Additionally, to explore the relationships between body composition, lower extremities power and torque with FSKT_MULT_. The main finding of the study was that HP had higher total and trunk lean mass while at the same time they had higher LJ and torque production. These results suggest that FSKT_MULT_ may be a valuable field test for coaches to evaluate the physical conditioning of athletes as well as to monitor body composition and lower extremities torque and power. Additionally, the study showed significant trivial-to-large correlation between performance in FSKT_MULT_ and body composition as well as with MT and PT during knee extension and flexion torques at 60°/s and 240°/s. Therefore, body composition may explain a portion of performance during FSKT_MULT_ whilst knee extension and flexion torque production may be a valuable laboratory test of performance for female taekwondo athletes. However, due to the exploratory design of the study, these results should be interpreted with caution.

The FSKT_MULT_ has previously been used as a field taekwondo test to evaluate the athlete’s repeated kicking ability during training [5], to compare athletes across different performance levels [9] and to classify athletes according to performance ranking [6]. In the present study, performance for the HP (FSKT_TOTAL_ = 89.5 ± 3.2 kicks) was classified between good and regular performers, whereas performance of the LP group (FSKT_TOTAL_ = 79.0 ± 3.0 kicks) fell within the regular performers category. Although weight category may not affect performance in FSKT_MULT_ for female athletes [6], total and trunk lean mass may significantly differentiate HP to LP taekwondo athletes. Lean mass has been shown to be a determinant of success in several power-based sports [8,27] and may distinguish high-level athletes from their lower-level counterparts [16]. Supporting this, a previous study in male surfers reported that athletes with greater muscle thickness produced higher power outputs and rate of force development [16]. Additionally, da Silva Santos et al. [9] demonstrated in 41 female taekwondo athletes that performance in FSKT_MULT_ effectively discriminates the competitive level from international/national to state/region level. Specifically, international/national athletes achieved more kicks during FSKT_BEST_ (20 vs. 19 kicks) and higher FSKT_TOTAL_ (91 vs. 86 kicks) differences that align with the findings of the present study. Taken together, these results suggest that lean mass may be a significant determinant factor distinguishing HP from LP female taekwondo athletes.

Athletes in the HP group produced higher isokinetic torques than those in the LP group. Significant differences emerged in peak torque for knee flexion and extension at 60°/s, and for knee extension at 240°/s. Moreover, HP athletes showed higher mean torque for knee flexion at 60°/s. These findings align with previous work comparing elite to sub-elite taekwondo athletes, where knee extension and flexion, along with hip internal and external rotation torques, were greater in elite performers during nine maximum velocity repetitions with the bandal-chagui kicking technique [7]. A more recent study also suggested that knee angular velocity combined with hip torques may best distinguish between elite and sub-elite taekwondo athletes, although performance was assessed again using only nine maximal bandal-chagui kicks [14]. In the present study, HP athletes appeared to benefit from the greater knee flexion and extension torque, particularly at slower velocities (60°/s), indicating that strength may be a key contributor to success in FSKT_MULT_. High-performer athletes also achieved higher LJ performance than LP athletes. A previous study in karate athletes suggests that horizontal movement patterns are highly relevant during training and competitions [28]. From a practical standpoint, LJ is a simple, low-cost field test that may offer coaches valuable insight into lower-extremity power in female taekwondo athletes. Contrariwise, CMJ variables did not differ between HP and LP athletes, consistent with earlier findings showing that CMJ does not reliably distinguish winners from losers in karate [29]. Consequently, LJ may serve as a more sensitive indicator of lower-extremity power in female taekwondo athletes.

Correlation coefficients between lean mass and FSKT_MULT_ ranged from trivial-to-large. Earlier studies in taekwondo athletes reported significant correlations between lean mass and FSKT_MULT_, although the athletes included in these studies were both males and females, which may have influenced the strength of the correlation [10,11]. In contrast, the present findings suggest that lean mass plays only a modest role in FSKT_MULT_ performance among female athletes. The study also revealed small-to-moderate correlations between BMD and FSKT_MULT_ (r = 0.240–0.513). Previous studies have shown that long-term training such as resistance training and plyometric exercises can meaningfully enhance bone remodeling [30,31]. Chronic taekwondo training may similarly contribute to higher BMD and reduced injury risk in both young [32] and older practitioners [33]. Additionally, negative correlations between BMD asymmetries and performance in set 5 of FSKT_MULT_ have been reported (r = −0.49; *p* = 0.04), further underscoring the relevance of BMD for performance [10]. Taken together, these results indicate that long-term taekwondo training may support BMD development in female athletes. However, because this is the first study to report these specific correlations, the findings should be interpreted cautiously.

Isokinetic knee extension and flexion showed small-to-large correlations with FSKT_MULT_ performance. Although these correlations were present at both testing velocities, correlations were stronger at 60°/s, and the right knee during both flexion and extension consistently demonstrated the closest relationship with performance. This pattern may be explained by leg preference, as all athletes selected the right leg in the Waterloo Footedness Questionnaire [23]. One previous study has examined the correlation between knee and hip torques with bandal-chagui performance, reporting that hip strength may be the dominant factor during maximal-velocity kicks [14]. However, to the authors’ knowledge, this is the first study to directly correlate FSKT_MULT_ performance with isokinetic knee extension and flexion torques; therefore, caution should be given to the interpretation of these results. Notably, lean mass and BMD showed moderate-to-very-large correlations with isokinetic torques at both testing velocities. Nevertheless, despite strong evidence for the relationship between lean mass and strength [34,35], no research has yet explored this association specifically in female taekwondo athletes. A study in nine elite male taekwondo athletes reported that leg lean mass was significantly associated with both rate of force development and maximum isometric peak force, findings that may suggest that greater lean mass supports rapid force production and maximal strength performance [13]. These findings suggest that increasing lean mass may enhance lower-body force production, potentially improve combat performance and reduce injury risk in female athletes.

In conclusion, the current exploratory study suggests that HP female taekwondo athletes appear to benefit from higher total and trunk lean mass, as well as greater lower-extremity power and torque as reflected in LJ performance and isokinetic knee extension and flexion torque. Lean mass and isokinetic knee extension-flexion torque may partially explain performance in FSKT_MULT_. However, since the nature of the current study is exploratory, these results should be interpreted cautiously by coaches and athletes. Additional research involving male athletes and international-level competitors is needed to draw more comprehensive and generalizable conclusions.

## 5. Limitations

The current study has some limitations. The relatively small sample size and the level of athletes who participated in the study may limit the generalization of the results to male taekwondo athletes or to athletes competing at the international level. Moreover, the age range and the unassessed maturation of female athletes may influence the results. Additionally, electromyographic activation and biomechanical variables that could influence FSKT_MULT_ performance were not assessed. Only leg extension and flexion torque were measured, with the absence of size-normalized values, which could have possibly controlled the level of the comparisons and correlations in this exploratory study. A conventional chest protector was used to measure FSKT_MULT_. Even so, a homogeneous sample of female taekwondo athletes participated in the study while the body composition and lower body torques were measured with DEXA and isokinetic, which strengthens the reliability of the results. This is a cross-sectional exploratory study; therefore, future studies should examine the relationship between body composition and lower extremities force production in male and female athletes as well as the possible differences that may occur following long-term taekwondo training on body composition and physical fitness in male and female taekwondo athletes.

## 6. Practical Applications

Coaches and strength and conditioning scientists should recognize that body composition quality, along with the ability to generate high muscle strength and power outputs, substantially influences performance in the FSKT_MULT_. High-performance female athletes are able to produce a greater number of kicks probably because they possess higher lean mass and demonstrate superior strength and power compared to lower-performance athletes. Training programs should therefore prioritize increasing lean mass, enhancing lower-limb strength, and maximizing muscle power. In addition, the strong correlations observed between lean mass and knee extensor and flexor torque suggest that lean mass not only contributes to athletic performance but also underpins strength development, which may translate into improved performance and reduce injury risk.

## Figures and Tables

**Table 1:** Differences in anthropometric characteristics, body composition, countermovement jump and long jump between high and low performers.

Variables	High Performers	Low Performers	t Value	*p* Value	Hedges g
Age (years)	17.6 ± 2.8	18.3 ± 3.0	0.615	0.545	0.233
Body Mass (kg)	59.9 ± 11.9	56.2 ± 7.7	−0.943	0.355	−0.358
Body Height (cm)	168.2 ± 4.6	164.2 ± 3.7	−2.402	0.024	−0.912
BMD (g/cm^2^)	1.182 ± 0.081	1.132 ± 0.056	−1.804	0.084	−0.685
Legs Lean Mass (kg)	13.3 ± 1.4	12.3 ± 1.2	−1.907	0.069	−0.724
Trunk Lean Mass (kg)	18.0 ± 1.6	16.7 ± 1.4	−2.196	0.038	−0.834
Total Lean Mass (kg)	38.8 ± 3.4	35.9 ± 3.1	−2.279	0.032	−0.866
Body Fat (%)	30.2 ± 7.6	31.2 ± 6.8	0.343	0.734	0.130
CMJ height (cm)	25.7 ± 3.8	25.4 ± 2.9	−0.170	0.867	−0.065
CMJ power (W)	2254.7 ± 588.7	2062.5 ± 402.1	−0.972	0.341	−0.369
CMJ power relative to mass (W/kg)	37.3 ± 3.7	36.5 ± 3.3	−0.629	0.535	−0.239
LJ (m)	1.98 ± 0.17	1.82 ± 0.14	−2.580	0.016	−0.980

BMD = bone mineral density, CMJ = countermovement jump, LJ = long jump.

**Table 2:** Correlation coefficients between body composition and variables from the multiple frequency speed of kick test.

	Total BMD	Legs Lean Mass	Trunk Lean Mass	Total Lean Mass	Percent Fat
FSKT_1SET_	0.363	0.393 *	0.380	0.416 *	−0.127
FSKT 2	0.513 **	0.524 **	0.555 **	0.571 **	−0.048
FSKT 3	0.457 *	0.370	0.481 *	0.470 *	−0.032
FSKT 4	0.379	0.211	0.167	0.230	−0.116
FSKT 5	0.347	0.169	0.289	0.283	−0.282
FSKT_TOTAL_	0.497 **	0.415 *	0.459 *	0.484 *	−0.128
FSKT_%KDI_	0.240	0.017	0.108	0.091	−0.013

FSKT_1SET_ = the number of kicks during the first set; FSKT 2 = the number of kicks during the second set; FSKT 3 = the number of kicks during the third set; FSKT 4 = the number of kicks during the fourth set; FSKT 5 = the number of kicks during the fifth set; FSKT_TOTAL_ = total kicks during the multiple frequency speed of kick test; FSKT %KDI = frequency speed of kick test percentage decrement index; * = *p* < 0.05, ** = *p* < 0.01. Trivial < 0.10; small < 0.10–0.29; moderate ≥ 0.30–0.49; large ≥ 0.50–0.69; very large ≥ 0.70–0.89; and nearly perfect ≥ 0.9.

**Table 3:** Correlation coefficients between the variables of multiple frequency speed of kick test and performance in knee extension and flexion torque during the 60°/s and 240°/s velocity testing for both right and left knees.

	FSKT_1SET_	FSKT 2	FSKT 3	FSKT 4	FSKT 5	FSKT_TOTAL_	FSKT_%KDI_
	**60°/s**						
MT Right Flexion	0.461 *	0.553 **	0.541 **	0.415 *	0.231	0.533 **	0.146
PT Right Flexion	0.446 *	0.600 **	0.621 ***	0.409 *	0.267	0.567 **	0.233
MT Left Flexion	0.448 *	0.490 *	0.494 *	0.321	0.228	0.482 *	0.055
PT Left Flexion	0.467 *	0.498 **	0.507 **	0.376	0.214	0.502 **	0.054
MT Right Extension	0.450 *	0.388	0.297	0.302	0.301	0.422 *	−0.046
PT Right Extension	0.447 *	0.389 *	0.321	0.334	0.342	0.441 *	0.005
MT Left Extension	0.272	0.286	0.346	0.173	0.187	0.313	0.024
PT Left Extension	0.396 *	0.389 *	0.412 *	0.218	0.252	0.409 *	−0.011
	**240°/s**						
MT Right Flexion	0.335	0.457 *	0.442 *	0.218	0.125	0.394 *	0.124
PT Right Flexion	0.329	0.505 **	0.481 *	0.220	0.101	0.410 *	0.164
MT Left Flexion	0.441 *	0.566 **	0.516 **	0.310	0.147	0.490 *	0.107
PT Left Flexion	0.357	0.451 *	0.465 *	0.272	0.113	0.410 *	0.105
MT Right Extension	0.186	0.352	0.292	0.056	0.187	0.270	0.145
PT Right Extension	0.294	0.444 *	0.412 *	0.120	0.187	0.362	0.127
MT Left Extension	0.183	0.302	0.206	−0.012	0.002	0.183	−0.025
PT Left Extension	0.276	0.334	0.262	0.052	0.020	0.244	−0.079

MT = Mean torque; PT = Peak torque; FSKT_1SET_ = the number of kicks during the first set; FSKT 2 = the number of kicks during the second set; FSKT 3 = the number of kicks during the third set; FSKT 4 = the number of kicks during the fourth set; FSKT 5 = the number of kicks during the fifth set; FSKT_TOTAL_ = total kicks during the multiple frequency speed of kick test; FSKT_%KDI_ = kick decrement index; * = *p* < 0.05; ** = *p* < 0.01; *** = *p* < 0.001. Trivial < 0.10; small < 0.10–0.29; moderate ≥ 0.30–0.49; large ≥ 0.50–0.69; very large ≥ 0.70–0.89; and nearly perfect ≥ 0.9.

**Table 4:** Correlation coefficients between body composition variables and knee extension and flexion mean and peak torque.

	Total BMD	Legs Lean Mass	Trunk Lean Mass	Total Lean Mass
	**60°/s**			
MT Right Flexion	0.599 **	0.723 ***	0.601 **	0.703 ***
PT Right Flexion	0.578 **	0.713 ***	0.596 **	0.684 ***
MT Left Flexion	0.540 **	0.732 ***	0.723 ***	0.760 ***
PT Left Flexion	0.578 **	0.720 ***	0.700 ***	0.738 ***
MT Right Extension	0.593 **	0.514 **	0.536 **	0.564 **
PT Right Extension	0.460 *	0.480 *	0.505 **	0.529 **
MT Left Extension	0.523 **	0.709 ***	0.691 ***	0.734 ***
PT Left Extension	0.444 *	0.715 ***	0.688 ***	0.736 ***
	**240°/s**			
MT Right Flexion	0.527 **	0.517 **	0.568 **	0.607 **
PT Right Flexion	0.522 **	0.583 **	0.600 **	0.642 ***
MT Left Flexion	0.544 **	0.523 **	0.503 **	0.561 **
PT Left Flexion	0.519 **	0.453 *	0.430 *	0.490 *
MT Right Extension	0.450 *	0.619 ***	0.693 ***	0.720 ***
PT Right Extension	0.401 *	0.732 ***	0.752 ***	0.786 ***
MT Left Extension	0.435 *	0.632 ***	0.682 ***	0.704 ***
PT Left Extension	0.400 *	0.749 ***	0.714 ***	0.762 ***

MT = Mean torque; PT = Peak torque; * = *p* < 0.05; ** = *p* < 0.01; *** = *p* < 0.001. Trivial < 0.10; small < 0.10–0.29; moderate ≥ 0.30–0.49; large ≥ 0.50–0.69; very large ≥ 0.70–0.89; and nearly perfect ≥ 0.9.

## Data Availability

The data are available from the corresponding Author following a reasonable request.

## Abbreviations

The following abbreviations are used in this manuscript:

HP	High performers
LP	Low performers
FSKT_MULT_	Multiple frequency speed of kick test
FSKT_BEST_	The highest number of kicks achieved in any set
FSKT_1SET_	The number of kicks during the first set of the multiple frequency speed of kick test
FSKT 2	The number of kicks during the second set of the multiple frequency speed of kick test
FSKT 3	The number of kicks during the third set of the multiple frequency speed of kick test
FSKT 4	The number of kicks during the fourth set of the multiple frequency speed of kick test
FSKT 5	The number of kicks during the fifth set of the multiple frequency speed of kick test
FSKT_TOTAL_	Total kicks during the multiple frequency speed of kick test
FSKT_%KDI_	Percentage kick decrement index during the multiple frequency speed of kick test
PT	Peak Torque
MT	Mean Torque
CMJ	Countermovement Jump
LJ	Standing long jump
DEXA	Dual-Energy X-Ray Absorptiometry
BMD	Bone mineral density
